# Identification of novel interacts partners of ADAR1 enzyme mediating the oncogenic process in aggressive breast cancer

**DOI:** 10.1038/s41598-023-35517-6

**Published:** 2023-05-23

**Authors:** Najat Binothman, Majidah Aljadani, Bandar Alghanem, Mohammed Y. Refai, Mamoon Rashid, Abeer Al Tuwaijri, Nouf H. Alsubhi, Ghadeer I. Alrefaei, Muhammad Yasir Khan, Sultan N. Sonbul, Fadwa Aljoud, Sultan Alhayyani, Rwaa H. Abdulal, Magdah Ganash, Anwar M. Hashem

**Affiliations:** 1 Department of Chemistry, College of Sciences and Arts, King Abdulaziz University, Rabigh, Saudi Arabia; 2 Vaccine and Immunotherapy Unit, King Fahad Medical Research Center, King Abdulaziz University Saudi Arabia, Jeddah, Saudi Arabia; 3 Medical Research Core Facility and Platforms (MRCFP), King Abdullah International Medical Research Center/King Saud bin Abdulaziz University for Health Sciences (KSAU-HS), King Abdulaziz Medical City (KAMC), National Guard Health Affairs (NGHA), Riyadh, Saudi Arabia; 4 Department of Biochemistry, College of Science, University of Jeddah, Jeddah, Saudi Arabia; 5 Department of AI and Bioinformatics, King Abdullah International Medical Research Center (KAIMRC), King Saud Bin Abdulaziz University for Health Sciences (KSAU-HS), King Abdulaziz Medical City, Ministry of National Guard Health Affairs, P.O. Box 22490, Riyadh, 11426 Saudi Arabia; 6 Medical Genomics Research Department, King Abdullah International Medical Research Center (KAIMRC), Ministry of National Guard Health Affairs (MNGH), Riyadh, Saudi Arabia; 7 Clinical Laboratory Sciences Department, College of Applied Medical Sciences, King Saud Bin Abdulaziz University for Health Sciences, Riyadh, Saudi Arabia; 8 Biological Sciences Department, College of Science & Arts, King Abdulaziz University, Rabigh, 21911 Saudi Arabia; 9 Department of Biology, College of Science, University of Jeddah, Jeddah, Saudi Arabia; 10 Department of Biology, Faculty of Science, King Abdulaziz University, Jeddah, 21589 Saudi Arabia; 11 Biochemistry Department, Faculty of Sciences, King Abdulaziz University, Jeddah, Saudi Arabia; 12 Experimental Biochemistry Unit, King Fahd Medical Research Center, King Abdulaziz University, Jeddah, Saudi Arabia; 13 Regenerative Medicine Unit, King Fahd Medical Research Centre, King Abdulaziz University, Jeddah, 21589 Saudi Arabia; 14 Department of Medical Microbiology and Parasitology, Faculty of Medicine, King AbdulAziz University, Jeddah, Saudi Arabia

**Keywords:** Cancer, Molecular biology

## Abstract

Triple-negative breast cancer (TNBC) subtype is characterized by aggressive clinical behavior and poor prognosis patient outcomes. Here, we show that ADAR1 is more abundantly expressed in infiltrating breast cancer (BC) tumors than in benign tumors. Further, ADAR1 protein expression is higher in aggressive BC cells (MDA-MB-231). Moreover, we identify a novel interacting partners proteins list with ADAR1 in MDA-MB-231, using immunoprecipitation assay and mass spectrometry. Using iLoop, a protein–protein interaction prediction server based on structural features, five proteins with high iloop scores were discovered: Histone H2A.V, Kynureninase (KYNU), 40S ribosomal protein SA, Complement C4-A, and Nebulin (ranged between 0.6 and 0.8). In silico analysis showed that invasive ductal carcinomas had the highest level of KYNU gene expression than the other classifications (p < 0.0001). Moreover, KYNU mRNA expression was shown to be considerably higher in TNBC patients (p < 0.0001) and associated with poor patient outcomes with a high-risk value. Importantly, we found an interaction between ADAR1 and KYNU in the more aggressive BC cells. Altogether, these results propose a new ADAR-KYNU interaction as potential therapeutic targeted therapy in aggressive BC.

## Introduction

Breast cancer (BC) presents a major challenge disease despite advancement in early detection and improved treatments due to its heterogeneity among different patients. Well-recognized studies have classified breast cancer tumors into four main intrinsic molecular subtypes with distinct prognostic and therapy implications^1^. Triple negative breast cancer (TNBC) is the most aggressive subtype of BC and is frequently classified as a subtype of basal-like BC^2,3^. Basal-like has unfavorable prognosis and aggressive tumor biology with limited therapy^4^. In contrast, Luminal A-subtype tumors have the most favorable prognosis and tumor biology with sufficient endocrine therapy^5^. Therefore, understanding the distinctive molecular mechanisms of cancer biology and development will facilitate discovery of new methods/mechanisms and targets for cancer therapy.

RNA editing is a prevalent post-transcriptional modification in the human transcriptome that alters RNA sequence without changing the genomic DNA sequence^6–8^. Despite the discovery of RNA editing more than 30 years ago, it brought a new understanding of genetic complexity that may impact various human diseases, including cancer^6–8^. In mammals, A-to-I RNA editing is the most widespread modification of editing events that involves editing specific nucleotide changes in double-stranded (ds) RNA increasing the diversity of transcriptome. This modification is mediated by a particular enzyme family designated as adenosine deaminase acting on RNAs (ADARs)^9–11^.

Adenosine Deaminase Acting on RNA 1 (ADAR1), a part of the ADARs enzyme family, catalyzes the irreversible conversion of adenosine to inosine by deamination within cellular dsRNA^9–11^. Several studies of ADAR1 expression levels and A-to-I RNA editing events alteration in cancer have been reported in the last few years. Indeed, Prestige’s studies discovered an up-regulation in RNA editing events as well as ADAR1 enzyme expression levels among 17 cancer types from 6,236 patient samples through using ‘The Cancer Genome Atlas’, a large-scale cancer sequencing project^13,14^. In BC, they found an increase in editing frequency and ADAR expression levels in 68 cancerous breast tissues compared to normal^13–15^. It has been found that higher ADAR1 expression was linked to tumor-infiltrating lymphocytes in patients with TNBC^16^. Recent finding has shown that ADAR1 promotes BC by regulating the cell cycle and DNA damage response^17^. ADAR1 was reported to be essential for TNBC transformation and tumorigenesis^18^. All above studies emphasized the vital role of ADAR1/A-to-I RNA editing process in BC progression and evaluated ADAR1 as the promising therapeutic target in BC. However, the mechanisms and proteins that regulate ADAR1/A-to-I RNA editing process remain mostly unidentified.

The study of protein–protein interaction (PPI) in the cellular milieu provides significant insight into a protein’s functions and mechanism. The usual way to capture PPI is experimental and labor-intensive^19^. Since, interacting protein pairs share a lot of structural, phylogenetic, and evolutionary features, different bioinformatics algorithms have been developed to exploit these features to predict PPI at a genome-wide scale^20^. With the surge of genome sequencing technology, the genomes of the organisms have been sequenced at a faster pace and thus gave birth to bioinformatics methods exploiting sequence features to predict PPI^21^. A wide variety of machine learning techniques have been used to predict PPI^22^. More recently, deep learning methods are also coming up for the prediction of PPI^23–26^.

In this work to gain insights into the interacting ADAR1 patterners, we used immunoprecipitation (IP) and mass spectrometry (MS)-based proteomics approaches to identify potential interacting proteins. We have identified several novel proteins interacting with ADAR1 in BC cells. In aggressive BC cells, five proteins were discovered using iLoop, a protein–protein interaction prediction website based on structural features: Histone H2A.V, Kynureninase (KYNU), 40S ribosomal protein SA, Complement C4-A, and Nebulin. For the first time, we discovered a novel physical interaction between ADAR1 and Kynureninase (KYNU) in aggressive BC cells. These findings could provide valuable resources for understanding cancer progression mechanisms and pave the way for new therapeutic targets in BC.

## Materials and methods

### Antibodies and reagents

Various antibodies were used, including anti-ADAR1 rabbit polyclonal antibody (Thermo Fisher), anti-Normal rabbit IgG (Sigma Aldrich), anti-GAPDH rabbit monoclonal antibody (Abcam), anti-KYNU rabbit polyclonal antibody (Thermo Fisher), anti-ADAR1 mouse monoclonal antibody (Thermo Fisher). Secondary antibodies were used, including goat anti-mouse (Abcam) and goat anti-rabbit IgG HRP (Thermo Fisher). For western blotting analysis, the primary antibodies dilutions were 1:1000 and GAPDH was used as the loading control. Secondary antibodies for western blotting analysis were diluted to 1:7000. Additional reagents used include pierce protein A/G agarose (Thermo Fisher), Protease inhibitor cocktail, EDTA-Free (100X) (Thermo Fisher), RIPA buffer, Pierce ip lysis buffer (Thermo Fisher), Sterile conical centrifuge tubes 15 ml (Thermo Fisher), cell culture flask 25 cm (Thermo Fisher).

### Cell culture

Dr. Alia Al-Amoudi's lab (King Abdulaziz University/King Fahad Medical Research Center, KSA) provided the MDA-MB-231 and MCF7 human BC cell lines. MDA-MB-231 cells were cultured in DMEM media (Thermo Fisher) containing 10% fetal bovine serum (FBS) (Thermo Fisher); while MCF7 cells were maintained in RPMI 1640 media (Thermo Fisher) containing 10% FBS (Thermo Fisher).

### Western Blotting

Western blot analysis was carried out by preparing total protein lysates with RIPA lysis buffer (Thermo Fisher) and a cocktail of protease inhibitors (Thermo Fisher). 8% SDS-PAGE, sodium dodecyl sulfate–polyacrylamide gel electrophoresis was used to resolve and separate 30 μg of total cellular proteins. Proteins are transferred to a nitrocellulose membrane. Membranes were blocked in 5% skimmed milk. The membranes were then incubated with the appropriate primary and secondary antibodies overnight at 4 °C.

### Immunoprecipitation and co-immunoprecipitation

IP lysis buffer (Thermo Fisher) and a protease inhibitor cocktail (Thermo Fisher) were used to generate protein lysates. 1 μg of anti-ADAR1 antibody was bound to 40 μl of protein A/G beads (Thermo Fisher) and incubated with 500 μl of total protein cell lysates. All the mixture incubated overnight at 4 °C. The beads’ washing step was achieved by using IP buffer. The beads were washed four times. Then, western blotting was accomplished by using SDS-PAGE gel and detected using appropriate primary and secondary antibodies.

### Immunofluorescence

Cell were seeded on coverslips until they reached between 80 to 90% confluency. The fixation step was accomplished by incubating the cell coated coverslips in 4% paraformaldehyde for 12 min at room temperature (RT). The permeabilization step was performed by incubating the cell coated coverslips in (0.2% Triton X-100) for 20 min at RT. Cells were then blocked for 45 min at RT with 2% goat serum. Cells were incubated overnight at 4 °C with a specific primary antibody (anti-ADAR1 mouse monoclonal antibody (Thermo Fisher). The primary antibody was diluted 1:200. The cells were then incubated for one hour at RT with the secondary antibody. Then, cells were incubated with the mounting media with the DAPI for few minutes. Cells were imaged using confocal microscopy (Zeiss LSM 700) fitted with a × 20 and × 40 objective.

### Tissue microarray

Tissue microarrays (TMA)/(BRC1021) were obtained from Pantomics Inc. The TMA includes 102 human BC patient’s samples with different information such as age, grade, stage and TNM. The H&E-stained slides were revised by a pathologist to confirm the BC tumor cases.

### Automated immunostaining

Immunohistochemistry (IHC) was performed in King Fahad Medical Research Center (KFMRC), IHC units, KSA following Automated immunostaining process^27^. The primary rabbit polyclonal antibody anti-ADAR1 (Thermo Fisher) with 1:1000 dilution was used and incubated at RT for one hour. The TMA slides were scanned using Grundium slide scanner.

### Scoring and evaluation of ADAR1 expression

ADAR1 protein expression’s evaluation was determine blindly of the clinicopathological features by using regular Nikon light microscope (40X magnification). The intensity of IHC staining was classified into four groups: (0): no/negative expression; (1 +): weak expression; (2 +): moderate, and (3 +): high expression. The scoring and evaluation was done in the KFMRC, IHC units, KSA^27^. The following formula using equation form was used to calculate both the intensity and the fraction of positively stained cells: I = 0xf0 + 1xf1 + 2xf2 + 3xf3. Following that, the staining was divided into two categories: (1) low (no/weak) expression, and (2) high (moderate/strong) expression^27^.

### Statistical analysis

Statistical analysis was accomplished using the SPSS® (IMB NY, USA) software packages (PASW Statistics for Windows, version 19). The Chi-square test was performed to analyze frequency tables and to calculate the significance of the correlations between the categorical variables. P-values < 0.05 were considered as statistically significant.

### Mass spectrometry

Protein was digested and desalted as described elsewhere^28^. In 0.1% formic acid, the dried peptide mixture was redissolved. The experiment was performed in King Abdullah University of Science and Technology, KSA, proteomics units as described elsewhere^29,30^.

## Results and discussion

### ADAR1 is highly expressed in infiltrating BC tumors than the benign tumors irrespective of the clinical-pathological parameters

**Figure 1:**
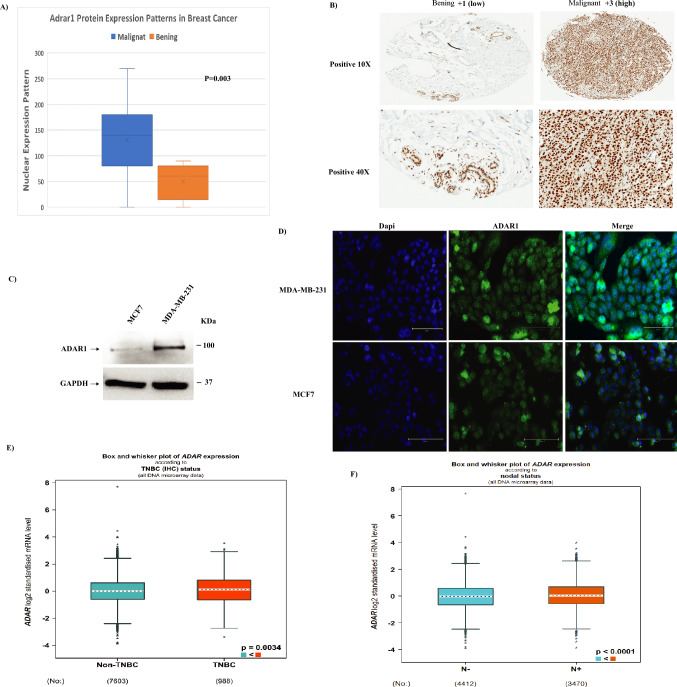
ADAR1 expression in breast cancer. (**A**) ADAR1 protein expression in malignant versus benign cases; (**B**) Positive immunohistochemical staining of ADAR1 in normal adjacent tissue, in situ and invasive breast cancer lesions (10X and 40X); (**C**) Immunoblot analysis of total cell lysates of MDA-MB-231 and MCF7 cells using antibodies against ADAR1and GAPDH; (**D**) Confocal immunofluorescence images of ADAR1 (green) and nucleus (Dapi) (blue) of breast cancer cells MDA-MB-231 and MCF7. Scale bars, 30 μm; (**E**) ADAR1 mRNA expression level in non-TNBC in comparison to TNBC tumor tissues using bcGenExMiner v4.8 database; (**F**) ADAR1 mRNA expression level in with or without lymph nodes involvements in tumor tissues using bcGenExMiner v4.8 database.

To study the clinical relevance of the role of ADAR1 in human BC, we explored the ADAR1 expression in human BC TMA. The TMA of 102 cases that include 97 BC cases and five normal/benign breast tissues. Importantly, we found that ADAR1 expression was higher (score above the mean value (= 131)) in 62/97 (64%) in BC cases compared to 0/5 (0%) benign cases (p = 0.003) (Fig. 1A,B). Then, we examined whether ADAR1 expression correlated with different clinicopathological parameters such as age and tumor grade and stage, we observed no significant correlation (Supplementary Table 1). Furthermore, we detect no statical correlation between ADAR1 protein expression and recognized biomarkers that define basic BC classification, including estrogen receptor (ER), progesterone receptor (PR) or human epidermal growth factor receptor 2 (HER2) tumor status (Supplementary Table 1). To validate these findings, we used a publicly available database including 5696 BC patients, Gene-Expression Miner v4.8 (bcGenExMiner v4.8), and found no significant association between ADAR1 mRNA levels and ER, PR, or HER2 (Supplementary Fig. 1A–C). Our results show that ADAR1 is highly expressed in infiltrating BC tumors than in benign tumors, regardless of the clinical pathological parameters.

To further investigate the role of ADAR1 in BC tumorigenesis, we examined the expression of ADAR1 in human BC cells, including MDA-MB-231 and MCF7 (represent Triple negative and Luminal A, respectively). Interestingly, we found higher ADAR1 protein expression in the more aggressive BC cell (MDA-MB-231) than the less aggressive cell (MCF7) (Fig. 1C). While similar patterns of ADAR1 localization, primarily nuclear and cytoplasmic, were observed in both cells (Fig. 1D). Moreover, ADAR1 mRNA expression was found to be significantly higher in TNBC patients (p < 0.003) and lymph node positive BC patients (LN +) (p < 0.0001) using bcGenExMiner v4.8 database (Fig. 1E,F). Collectively, these findings suggest that ADAR1 is highly expressed in aggressive BC cells and may play a role in aggressive BC behavior.

### Analysis of endogenous ADAR1 protein interactors in aggressive BC cells, TNBC (MDA-MB-231)

**Figure 2:**
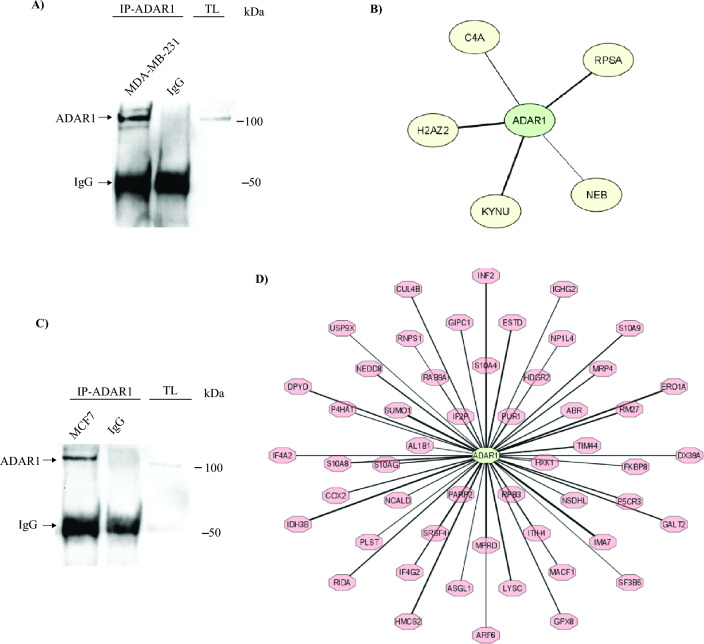
ADAR1 interaction partners in breast cancer cells. (**A**) MDA-MB-231 cells were lysed, and immunoprecipitations were performed using a rabbit polyclonal antibody against ADAR1 or control normal rabbit IGg. Western blotting was carried out using an antibody against ADAR1; (**B**) The interaction network of ADAR1. The ADAR1 interactome in MDA-MB-231 cell line was generated based on structural features using iLoops and visualized in Cytoscape. The thickness of the edge correlates with iLoops score; (**C**) MCF7 cells were lysed and immunoprecipitations were performed using a rabbit polyclonal antibody against ADAR1 or control normal rabbit IGg. Western blotting was carried out using an antibody against ADAR1; (**D**) The interaction network of ADAR1. The ADAR1 interactome in MCF-7 cell line was generated based on structural features using iLoops and visualized in Cytoscape. The thickness of the edge correlates with iLoops score.

To discover the ADAR1 protein partner, we first performed an IP assay to pull down the ADAR1 enzyme in the TNBC MDA-MB-231 cells. Normal IgG pull-down was included as negative control and total lysate as the positive control. Western blotting was carried out using primary antibody against ADAR1. Indeed, we found enrichments (bands) of ADAR1 in MDA-MB-231 cells (Fig. 2A), whereas the band was not present in the IgG control. As a result, ADAR1 IP from BC cells was successful.

Next, to identify the ADAR1 proteins interaction we performed MS analysis including two biological replicates and 3 technical replicates. The preparation of samples including digestion/desalted and MS analysis was performed by King Abdullah University of Science and Technology, KSA. The MS data were analyzed using Proteome discoverer (version2.5). The Uniprot human database (released June 25, 2021) was used for the database search. Hundreds of proteins were identified as shown in the table S2 (Supplementary Table 2). The list of identified proteins was then filtered manually by removing contaminates proteins and keeping only these identified on both biological replicates. Furthermore, only significant differentially expressed proteins with *p* value < 0.05 and fold change > 2 compared to control sample (IgG) were included. These filtration steps generated a total 13 proteins for MDA-MB-231 (Supplementary Table 3).

To further gain insights into the understanding of ADAR1 interact patterners overall, we explore ADAR1 physical interact partner to a publicly available database STRING, a biological database of protein–protein interaction prediction network (STRING DB V 10.5-http://string-db.org/). We found seven proteins (Supplementary Fig. 2): UPF1, DICER1, ILF3, RBMX, ELAVL1, AXIN1 and HDLBP interacted with ADAR based on experimental data such as IP assay in different kind of diseases and posttranscriptional regulatory process^31–34^. However, none of these interactions have been studied in BC. Consequently, we found no common proteins between our finding and STRING result. Therefore, we used iLoops, a protein–protein interaction prediction server based on structural features, to investigate the interactome of ADAR1 in the aggressive BC cell line (MDA-MB-231). As a result, five of the 13 proteins were found to interact with the ADAR1 enzyme with a high iloop_score (ranged between 0.6 and 0.8). As shown in Fig. 2B, these proteins are Histone H2A.V, KYNU, 40S ribosomal protein SA, Complement C4-A, and Nebulin.

To further confirm our results, we did the same IP assay and MS experiment followed by the same analysis for the less aggressive BC cell (luminal A subtype), MCF7. As a result, 82 proteins with a high iloop score were found to interact with the ADAR1 enzyme (Fig. 2C,D, and Supplementary Table 4). Interestingly, none of the 82 proteins in MCF7 shared any similarities with the five proteins identified in MDA-MB-231 cells. These results indicate that these five proteins, Histone H2A.V, KYNU, 40S ribosomal protein SA, Complement C4-A and Nebulin, are specific for interacting with ADAR1 enzyme in TNBC, which is define as the most aggressive subtypes of BC.

### Implication of Histone H2A.V, KYNU and 40S ribosomal protein SA with poor patient outcome in BC

**Figure 3:**
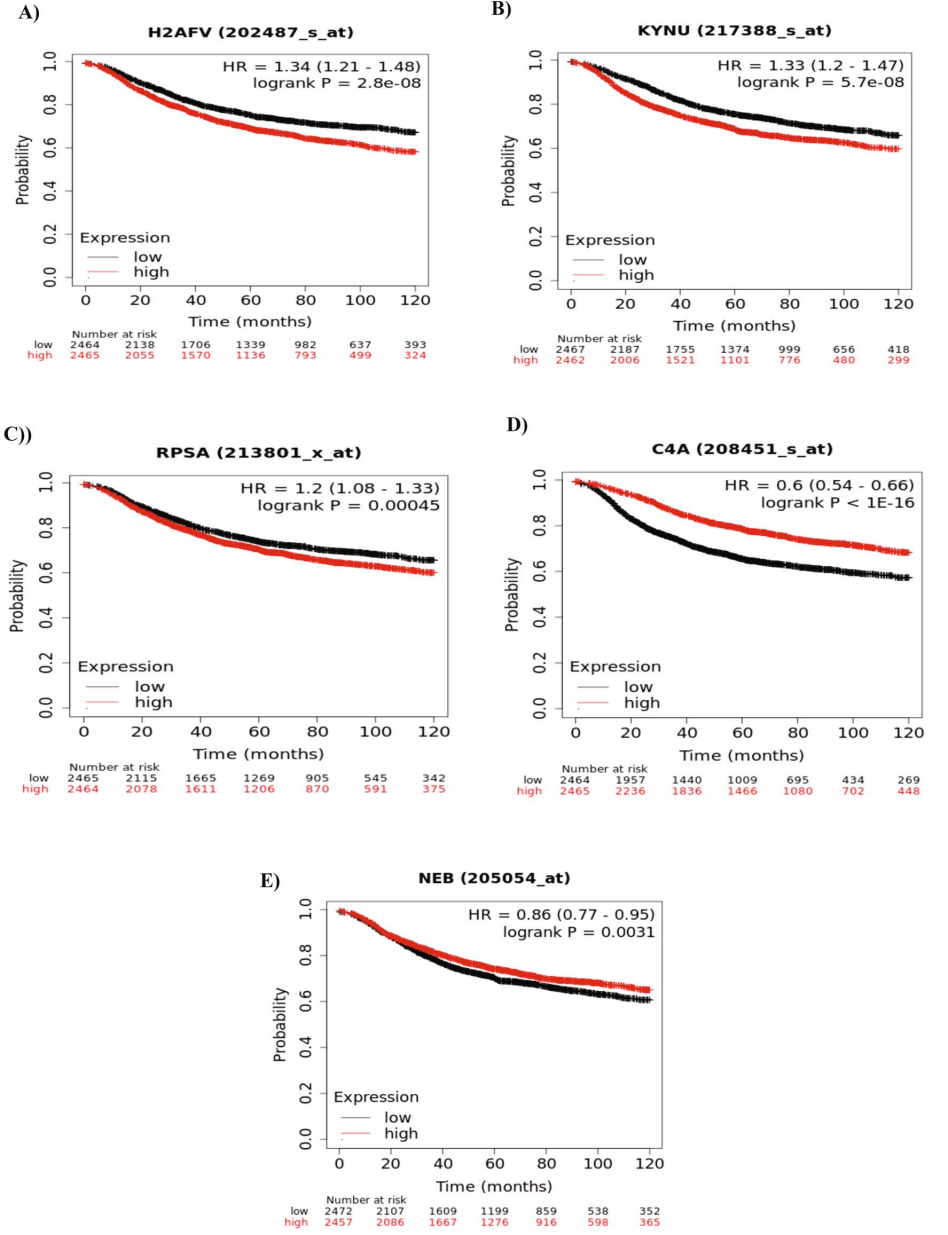
H2AFV, KYNU, RPSA, C4A and NEB expression in breast cancer. (**A**–**E**) Kaplan–Meier survival curves of H2AFV, KYNU, RPSA, C4A and NEB gene expression in association with patient outcome (4934 KM-plotter database) using RFS as an end point.

Next, to validate the role of Histone H2A.V, KYNU, 40S ribosomal protein SA, Complement C4-A and Nebulin in BC progression, we examine the mRNA levels and patient outcomes in relation to relapse free survival (RFS). Kaplan–Meier plotter, an extensive gene profiling database including more than 5 thousand BC cases, was used. Interestingly, high mRNA levels of Histone H2A.V, KYNU, and 40S ribosomal protein SA were found to be significantly associated with decreased RFS and an unfavorable prognosis with high-risk value (1.34, 1.33 and 1.2, respectively) (Fig. 3A–C). On the other hand, Complement C4-A and Nebulin showed less risk factor (0.66 and 0.86, respectively) and were associated with increased RFS and favorable prognosis (Fig. 3D,E).

**Figure 4:**
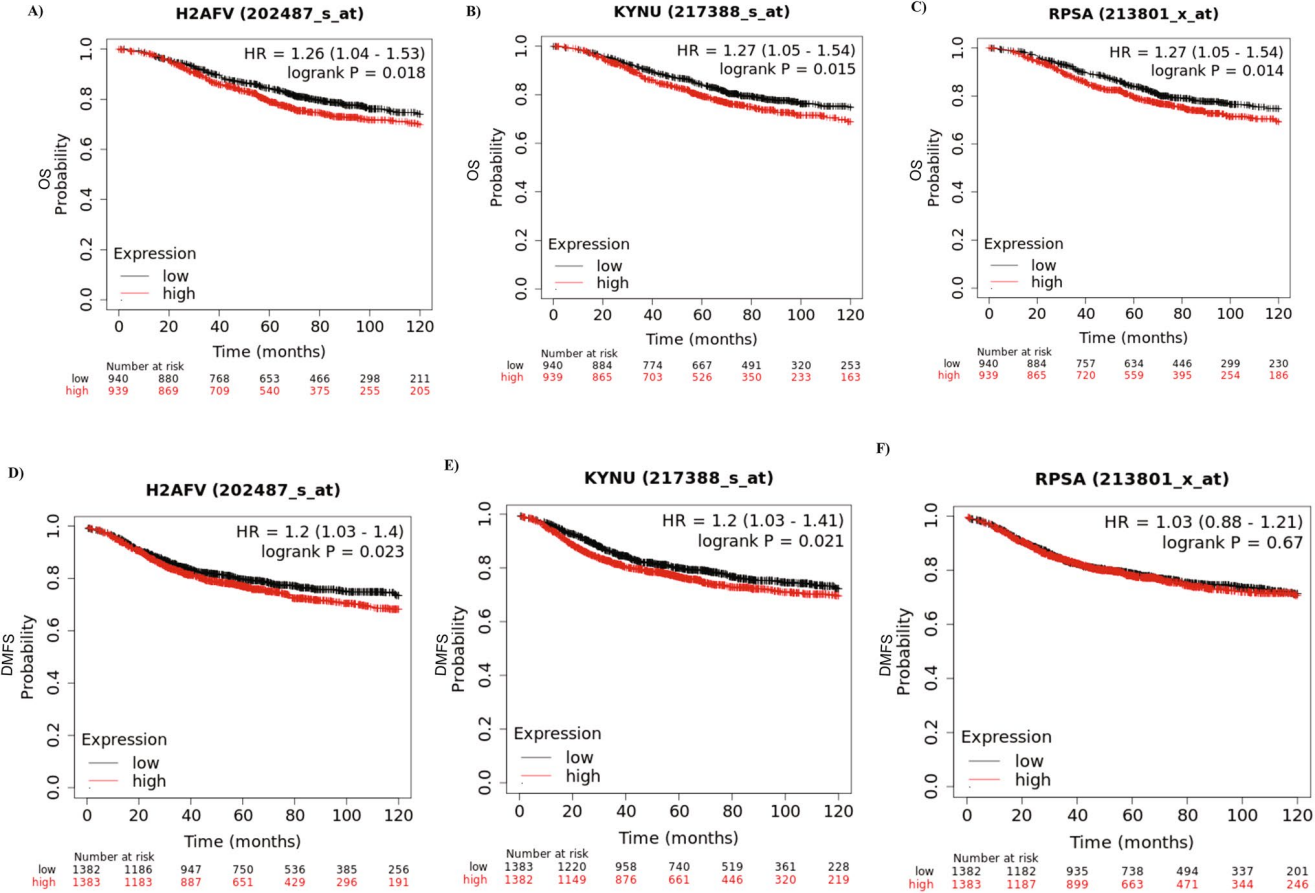
H2AFV, KYNU and RPSA gene expression in breast cancer. (**A**–**F**) Kaplan–Meier survival curves of H2AFV, KYNU, and RPSA gene expression in association with patient outcome (1880 & 2767 KM-plotter database) using OS & DMFS, respectively as an end point.

Following the above results, patients with higher Histone H2A.V, KYNU, and 40S ribosomal protein SA gene expression levels exhibited significantly shorter overall survival (p = 0.018, 0.015, and 1.014, respectively) and distant metastasis free survival (Fig. 4A–F). Overall, these results indicate that the top three predicted ADAR1 interacted proteins (Histone H2A.V, KYNU, and 40S ribosomal protein SA) are linked to an aggressive BC phenotype.

### Expression of Histone H2A.V, KYNU, and 40S ribosomal in BC patients

**Figure 5:**
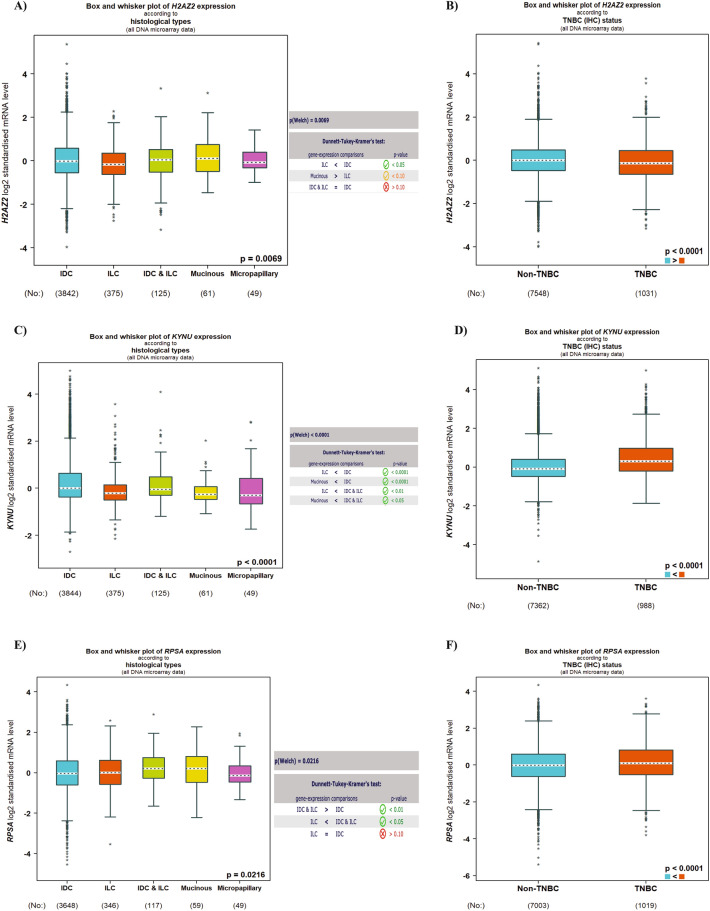
H2AFV, KYNU and RPSA gene expression in breast cancer according to histological type. (**A–F**) mRNA expression level of H2AFV, KYNU and RPSA according to histological type and mRNA expression level in non-TNBC in comparison to TNBC tumor tissues using bcGenExMiner v4.8 database.

Next, to determine the involvement in BC tumorigenesis, we examined the expression level of the Histone H2A.V, KYNU and 40S ribosomal protein SA using a publicly available database including 5696 BC patients, Gene-Expression Miner v4.8 (bcGenExMiner v4.8). Based on diverse histological classes, Histone H2A.V mRNA expression was significantly higher in invasive ductal carcinomas (IDC) than in invasive lobular carcinomas (p = 0.05). However, there was no significant difference between IDC and other classifications such as mucinous carcinoma (Fig. 5A). IDC, well-known as infiltrating ductal carcinoma, is the most invasive type which can spread to lymph nodes or blood vessels and metastasize throughout the body. We found that Histone H2A.V mRNA expression levels were higher in non-TNBC patients based on TNBC/IHC status (Fig. 5B). Interestingly, in comparison to other classifications, invasive ductal carcinomas had the highest level of KYNU gene expression, followed by invasive lobular carcinoma (p < 0.0001) and mucinous carcinomas (p < 0.0001) (Fig. 5C). Moreover, KYNU mRNA expression was found to be significantly higher in TNBC patients (p < 0.0001) (Fig. 5D).

Next, we performed the same analysis on 40S ribosomal expression and found that IDC had higher mRNA levels but no significant difference from other classifications (Fig. 5E). 40S ribosomal expression was to be higher in TNBC patients (p < 0.0001) (Fig. 5F). Our findings show that upregulation of KYNU expression is the most meaningfully associated with IDC, TNBC, and poor prognosis patient outcomes.

### Detection of ADAR1 and KYNU interaction in aggressive BC cell

**Figure 6:**
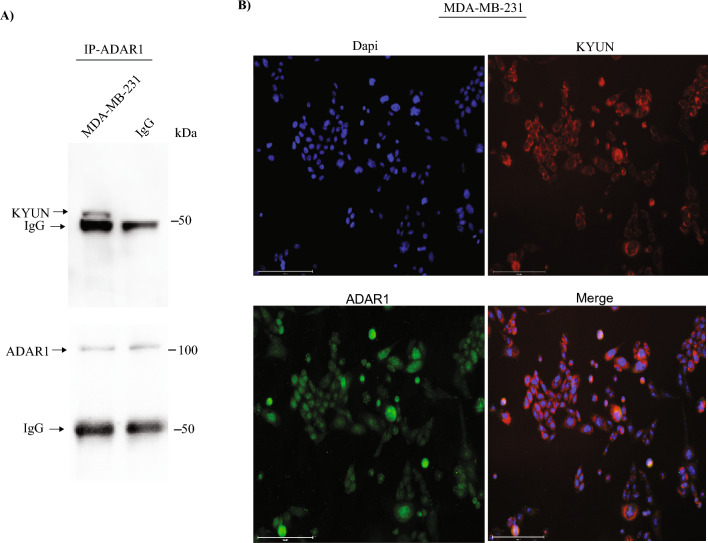
KYNU and ADAR1 interaction in aggressive breast cancer cell and subcellular localization. (**A**) MDA-MB-231 cell were lysed and immunoprecipitations were performed using a rabbit polyclonal antibody against ADAR1 or control normal rabbit IGg. Western blotting was carried out using a rabbit polyclonal antibody against KYNU (upper panel). Membrane was reprobed using a rabbit polyclonal against ADAR1 (lower panel); (**B**) Confocal immunofluorescence images of KYUN (red), ADAR1 (green) (using mouse monoclonal antibody against ADAR1) and nucleus (Dapi) (blue) of breast cancer cell MDA-MB-231. Scale bars, 150 μm.

KYNU is enzymes that play a vital role in tryptophan metabolism through the degradation of intermediate 3'-hydroxy-kynurenine metabolites to produce 3‐hydroxyanthranilic acid. KYNU contributes to the biosynthesis of NAD cofactors from tryptophan via the kynurenine pathway. KYNU has been reported being associated with various diseases such as metabolic, neurological, cardiac and renal disease. The role of KYNU in BC development has not been illustrated and is mostly unclear. Recently, it was found that KYNU promotes tumor cell invasion via CD44^35^. These finding suggest that the interaction between ADAR1 and KYNU may play a role in ADAR1 oncogenic pathway in aggressive BC. Indeed, we found interaction between ADAR1 and KYNU in MDA-MB-231 cell (Fig. 6A). Next, we evaluate the subcellular localization of KYNU. Interestingly, KYNU was found to be perinuclear, whereas ADAR1 was found in both the nuclear and cytoplasm (Fig. 6B). These findings suggest that the ADAR1-KYNU interaction is important in aggressive BC cells and could be a potential therapeutic target. However, more research is needed to understand the mechanism underlying the ADAR1-KYNU interaction's role in BC.

## Conclusion

This study revealed a novel interaction partner ADAR1 enzyme that may play a critical role in mediating the oncogenic process of ADAR1 in aggressive BC. One of the top potential candidates, KYNU, was found to be expressed and is the most meaningfully correlates with invasive ductal carcinomas, TNBC, and poor prognosis patient outcomes. Interestingly, we found an interaction between ADAR1 and KYNU in MDA-MB-231 cells, with perinuclear localization of KYNU, which may play an important role in the aggressiveness of BC. Overall, these findings contribute to a better understanding of the mechanisms underlying cancer progression and the identification of new therapeutic targets in breast cancer.

## Supplementary Information

Supplementary Information 1.

Supplementary Table 2.

Supplementary Table 4.

## Data Availability

The datasets used and/or analysed during the current study available from the corresponding author on reasonable request. The data that support the findings of this study are available from KAUST but restrictions apply to the availability of these data, which were used under license for the current study, and so are not publicly available. Data are however available from the authors upon reasonable request and with permission of KAUST.

## Abbreviations

BC: Breast cancer
TNBC: Triple negative breast cancer
ADARs: Adenosine deaminases acting on RNA
KYNU: Kynureninase
FBS: Fetal bovine serum
IP: Immunoprecipitation
RT: Room temperature
IHC: Immunohistochemistry
TMA: Tissue microarrays
ER: Estrogen receptor
PR: Progesterone receptor
HER2: Human epidermal growth factor receptor 2
MS: Mass spectrometry
RFS: Relapse free survival
IDC: Invasive ductal carcinomas
