# Supplementary material for: Identification of novel interacts partners of ADAR1 enzyme mediating the oncogenic process in aggressive breast cancer
Source: Sci Rep. 2023 May 23;13:8341. doi: 10.1038/s41598-023-35517-6 (PMC10206070; doi:10.1038/s41598-023-35517-6)
Supplement: Supplementary file 1 — Supplementary Information 1. [file 41598_2023_35517_MOESM1_ESM.docx]

**Supplementary information for:**

Identification of Novel Interacts Partners of ADAR1 Enzyme Mediating the Oncogenic Process in Aggressive Breast Cancer

Najat Binothman^1*^, Majidah Aljadani^1^, Bandar Alghanem^2^, Mohammed Y Refai^3^, [Mamoon Rashid](https://pubmed.ncbi.nlm.nih.gov/?term=Rashid+M&cauthor_id=35879805)^4^, Abeer Al Tuwaijri^5,6^, Nouf H. Alsubhi^7^, Ghadeer I. Alrefaei^8^, Muhammad Yasir Khan^9^, Anwar M Hashem^9,10^, Sultan N. Sonbul^11,12^, Fadwa Aljoud^13,14^, Sultan Alhayyani^1^, Rwaa H. Abdulal^9,15^, Magdah Ganash^15^

^1^Department of Chemistry, College of Sciences and Arts, King Abdulaziz University, Rabigh, Saudi Arabia

**^*^Corresponding author**

Dr. Najat Binothman

**Contents:**

- **Supplementary Figure 1**
- **Supplementary Figure 2**
- **Supplementary Table 1**
- **Supplementary Table 2**
- **Supplementary Table 3**
- **Supplementary Table 4**
- **The original images of the western blotting gels**

**
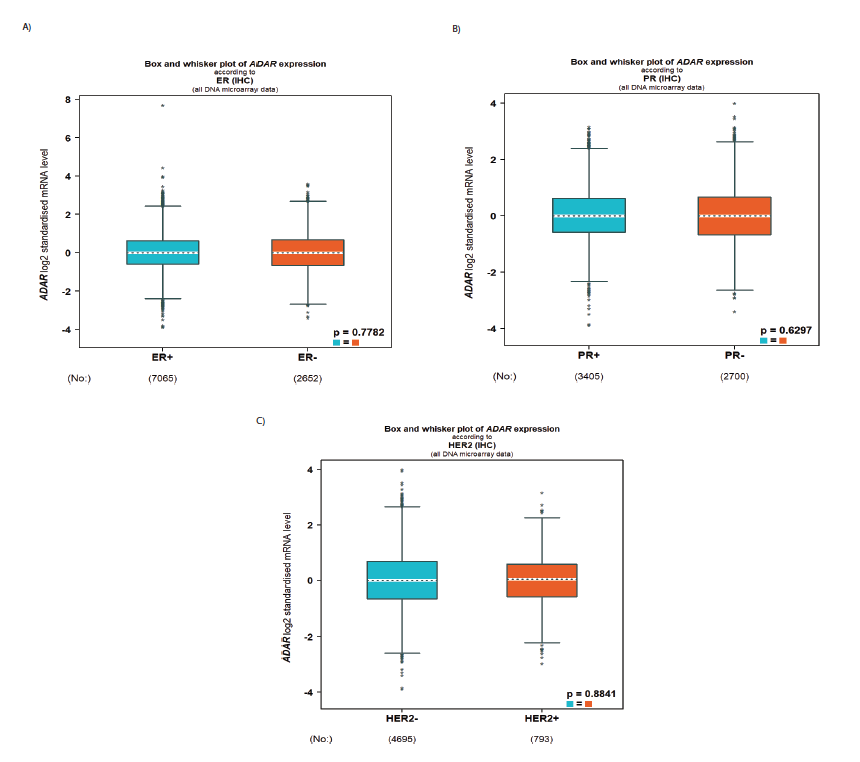
**

**Supplementary Figure 1. The association of ADAR1 mRNA levels with ER, PR and HER2 status**

**
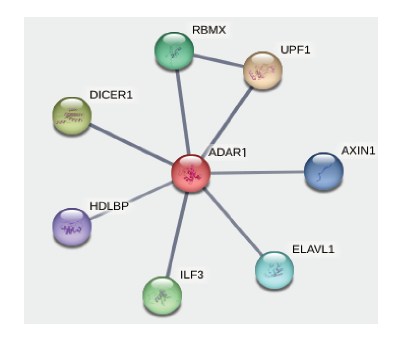
**

**Supplementary Figure 2. ADAR1 network proteins interaction using publicly available database (STRING).**

**Supplementary Table 1. Clinicopathological features of the human breast cancer tissue microarrays:** Associations between ADAR1 protein expression and different clinicopathological parameters including age, histopathology type, tumor grade, tumor stage, Androgen receptor (AR) score, Progesterone receptor (PR) score, HER2 score and Ki67 score.

**
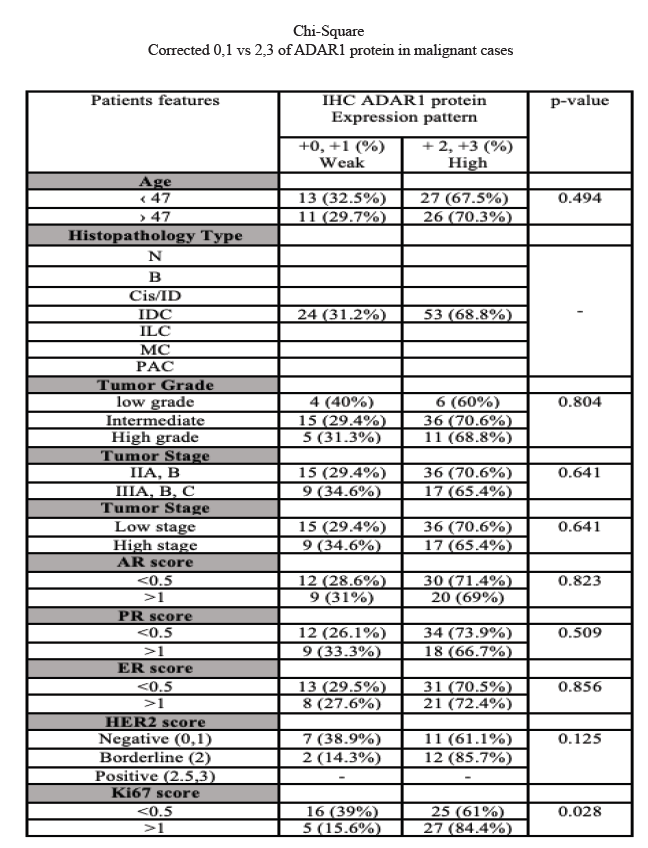
**

**Supplementary Table 2. The MS data of IP-ADAR1 in MDA-MB-231 using Proteome discoverer (version2.5):** Identification of numerous ADAR1 proteins interaction in aggressive breast cancer cell using MS analysis/Proteome discoverer (version2.5). The preparation of samples including digestion/desalted and MS analysis. (Note: due to the large data in the Excel sheet, we provide the table as hyper link)

**Supplementary Table 3. Identification of significant differentially expressed proteins:** The list of identified proteins by filtering them manually and removing contaminates proteins and keeping only these identified on both biological replicates. only significant differentially expressed proteins with *p* value < 0.05 and fold change > 2 compared to control sample (IgG) were included.

**
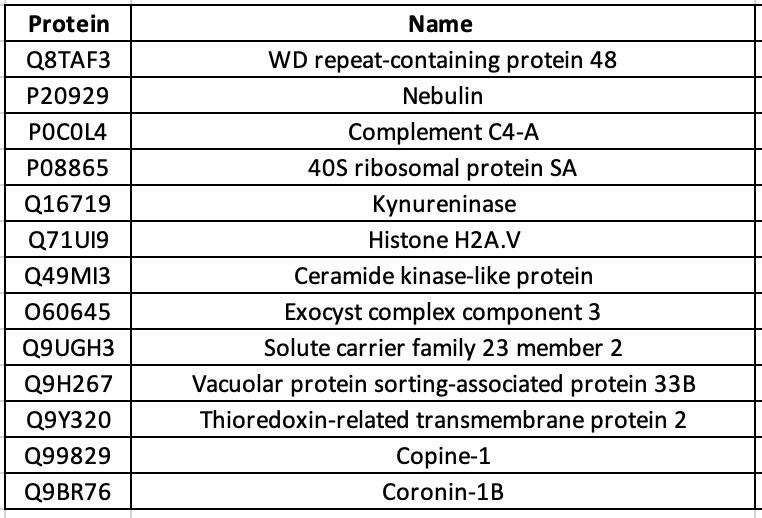
**

**Supplementary Table 4. The MS data of IP-ADAR1 in MCF7 using Proteome discoverer (version2.5):** Identification of numerous ADAR1 proteins interaction in less aggressive breast cancer cell using MS analysis/Proteome discoverer (version2.5). The preparation of samples including digestion/desalted and MS analysis. (Note: due to the large data in the Excel sheet, we provide the table as hyper link).

**- The original images of the western blotting gels including the invert images:**

1. Figure 1 (C):

(WB of ADAR1)

**MCF7 MDA-231 MCF7 MDA-**231 invert image


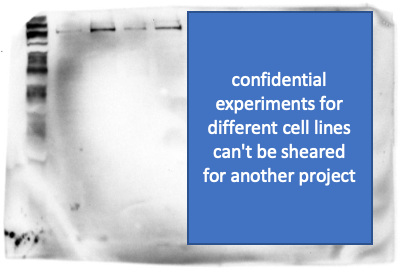

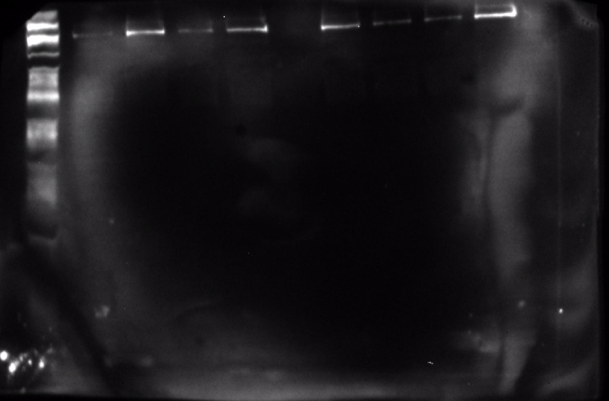


(WB of GAPDH)

**MCF7 MDA-231 MCF7 MDA-231**


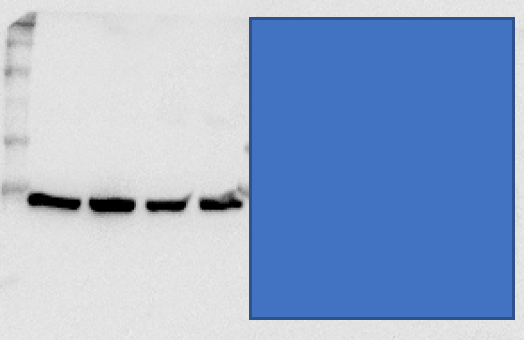

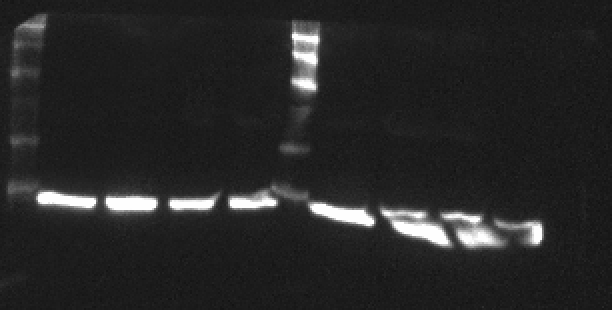


Figure 2 (A & C): (IP ADAR1, western bolt ADAR1)

MDA-MB-231 MCF7

**
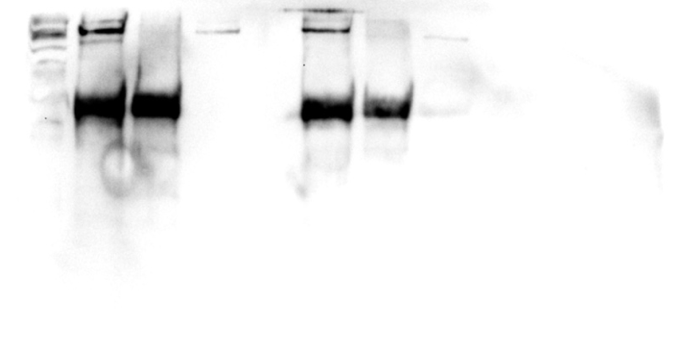
**
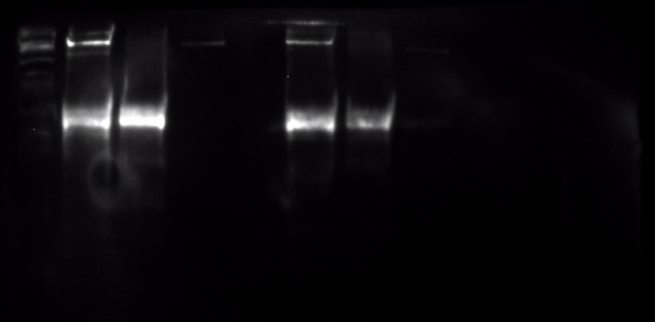


1. Figure 6 (A):

Co-IP:

(IP ADAR1, western bolt of KYNU)

MDA-MB-231

**
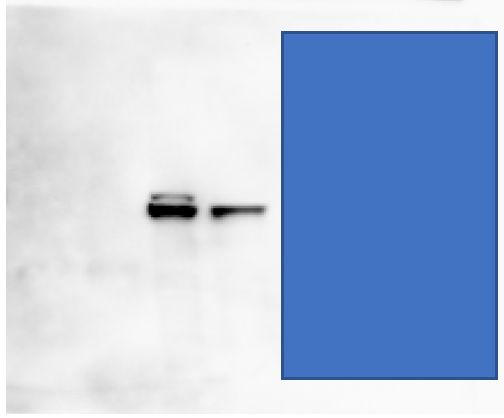

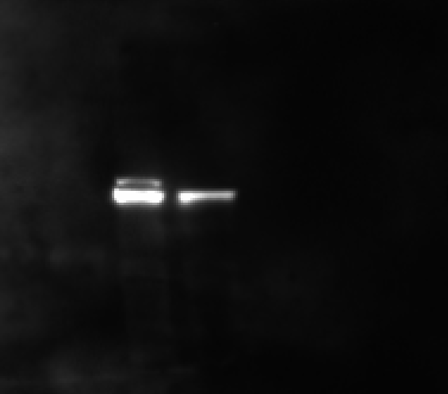
**

(IP ADAR1, western bolt ADAR1)

MDA-MB-231

**
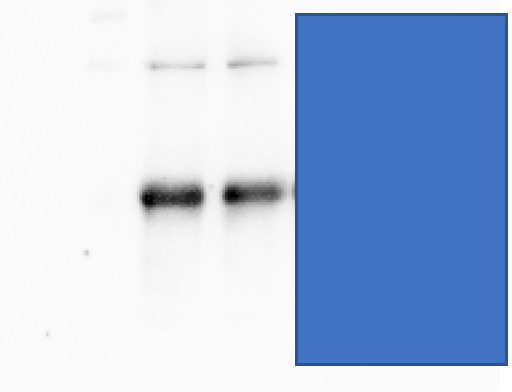

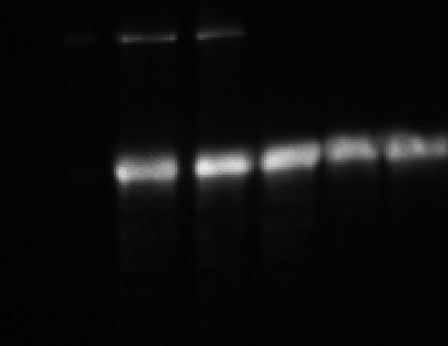
**
